# Hospital Meetings

**Published:** 1905-03-11

**Authors:** 


### HOSPITAL MEETINGS.

### QUEEN CHARLOTTE'S LYING-IN HOSPITAL.

The annual meeting of governors and subscribers of Queen Charlotte's Hospital was held last week, Mr. F. W. Hunt presiding. The chairman referred to the great loss the hospital had sustained by the death of the Earl of Hardwicke, who had ably filled the office of chairman of the committee for over six years. The report stated that 1,456 women had been delivered in the hospital during 1904, and 1,754 had been attended and nursed in their own homes. The ordinary expenditure had amounted to £5,829, whereas the ordinary income was £5,093 only, including £500 from King Edward's Hospital Fund and £683 proceeds of the concert given by Madame Melba in aid of the hospital. The legacy under the will of the late Edmond Dresden, to form the "Dresden Assistance Fund" (to be applied solely for the purpose of assisting patients on leaving the hospital) had been received, and would enable the committee to help a considerable number of necessitous patients. To the Convalescent Home 129 patients had been admitted. The Princess Christian had presided at the annual meeting of the Mothers' Aid Society, and had been pleased to say that she would visit the wards of the hospital on a subsequent occasion. The important work of the Midwifery Training School had made satisfactory progress. In order to provide for the increased number of patients and nurses, the nurses' home had been enlarged by purchasing three houses adjoining the home, and three rooms in the hospital had been converted into wards—increasing the total accommodation for patients to 69 beds. An expenditure of over £4,000 had thereby been incurred, towards which the committee earnestly appealed for contributions.

